# Post-partum Women’s Anxiety and Parenting Stress: Home-Visiting Protective Effect During the COVID-19 Pandemic

**DOI:** 10.1007/s10995-022-03540-0

**Published:** 2022-09-24

**Authors:** Elisa Roberti, Roberta Giacchero, Serena Grumi, Giacomo Biasucci, Laura Cuzzani, Lidia Decembrino, Maria Luisa Magnani, Mario Motta, Renata Nacinovich, Camilla Pisoni, Barbara Scelsa, Livio Provenzi, MOM-COPE study group, Lilia Altieri, Pietra Benedetta, Elisa Bettiga, Renza Bonini, Renato Borgatti, Anna Cavallini, Rossana Falcone, Elisa Fazzi, Barbara Gardella, Paola Guerini, Simona Orcesi, Dario Pantaleo, Federico Prefumo, Roberto Previtali, Laura Riva, Arsenio Spinillo, Patrizia Vergani, Maria Grazia Viganò

**Affiliations:** 1 Developmental Psychobiology Lab, IRCCS Mondino Foundation, via Mondino 2, 27100 Pavia, Italy; 2 Department of Pediatrics, ASST Lodi, Lodi, Italy; 3 Obstetrics and Gynecology Unit, Guglielmo da Saliceto Hospital, Piacenza, Italy; 4 ASST Pavia, Unità Operativa di Pediatria e Nido, Ospedale Civile di Vigevano, Vigevano, PV Italy; 5 ASST Pavia, Unità Operativa di Pediatria e Nido, Ospedale Civile di Voghera, Voghera, PV Italy; 6 Department of Neonatology and Neonatal Intensive Care Unit, ASST Spedali Civili, Brescia, Italy; 7 Clinic of Child and Adolescent Neuropsychiatry, San Gerardo Hospital, ASST Monza, Monza, Italy; 8 Department of Medicine and Surgery, University of Milano Bicocca, Milan, Italy; 9 Neonatal Intensive Care Unit, IRCCS Foundation Policlinico San Matteo, Pavia, Italy; 10 Unit of Pediatric Neurology, Buzzi Children’s Hospital, Milan, Italy; 11 Department of Brain and Behavioral Sciences, University of Pavia, Pavia, Italy

**Keywords:** Anxiety, Parenting stress, COVID-19, Home-visiting, Mothers

## Abstract

**Objectives:**

The COVID-19 pandemic resulted in a particularly adverse and stressful environment for expecting mothers, possibly enhancing feelings of anxiety and parenting stress. The present work assesses mothers' anxiety levels at delivery and parenting stress after 3 months as moderated by home-visiting sessions.

**Methods:**

Women (n = 177) in their second or third trimester of pregnancy during the COVID-19 lockdown were enrolled in northern Italy and split into those who did and did not receive home visits. After 3 months, the association between anxiety at delivery and parenting stress was assessed with bivariate correlations in the whole sample and comparing the two groups.

**Results:**

Higher anxiety at birth correlated with greater perceived stress after 3 months. Mothers who received at least one home-visiting session reported lower parenting stress at 3 months than counterparts who did not receive home visits.

**Conclusions for Practice:**

The perinatal period is a sensitive time window for mother-infant health, especially during a critical time like the COVID-19 pandemic. We suggest that home-visiting programs could be beneficial during global healthcare emergencies to promote maternal well-being after delivery.

## Significance

*What is already known on this topic* Adverse conditions, such as the pandemic, during developmental windows of heightened plasticity increase the risk of maladaptive stress responses. Both pregnancy and the postnatal period represent a time of exceptional environmental sensitivity for mothers and infants. Home-visiting programs may be beneficial in increasing mothers’ self-confidence, protecting them from developing severe affective symptoms and parenting stress.

*What this study adds* Home-visiting reduces parental stress, protecting the dyadic interactive aspects of the mother-infant relationship. Home visits are specifically beneficial for mothers who present high anxiety levels after delivery and provide a social resource, contrasting the isolation and trauma experienced during the COVID-19 pandemic. These results have both research and clinical implications and may inform healthcare policies for mother-infant health.

## Introduction

The COVID-19 pandemic is an unprecedented global healthcare emergency and a collective traumatic experience (Bo et al., 2021; Kanzler & Ogbeide, 2020). The high rates of SARS-CoV-2 contagion, its rapid spread, the partial availability of effective treatments or vaccines, together with social distancing and economic challenges implied by mitigation and containment strategies, contribute to relevant distress and raise concerns for public health (Graffigna et al., 2021; Ozamiz-Etxebarria et al., 2020). Previous literature has extensively demonstrated that individuals exposed to such intense adverse conditions during developmental windows of heightened plasticity (Kim, 2016) may be susceptible to developing maladaptive stress responses and affective symptoms (Davis & Narayan, 2020; Pluess & Belsky, 2011). Consistently, it is of paramount importance to study the impact of pandemic-related stress on the health of individuals who are experiencing sensitive periods and the protective role of preventive and supportive interventions (Horesh & Brown, 2020; Provenzi & Tronick, 2020).

Both pregnancy and the postnatal (i.e., the first 3 months after birth) period represent an exceptionally sensitive time for mothers and infants. Not surprisingly, reports of affective symptoms are not uncommon in the post-partum. During the pre-pandemic period (i.e., before March 2020, when lockdown began in Italy due to the outbreak of the coronavirus disease), the prevalence of full-blown depressive symptomatology was estimated in 10–15% of women after delivery in a meta-analysis of 291 studies from 56 countries (Hahn-Holbrook et al., 2018); whereas clinically relevant anxiety symptoms were observed in 10% of mothers in a meta-analysis of 102 studies from 34 countries (Dennis et al., 2017). Meta-analytic evidence suggested that levels of post-natal depression in mothers have increased during the present pandemic (Hessami et al., 2020). Nonetheless, anxiety is the most reported psychological symptom in pregnant women and new mothers in different countries hit by the COVID-19 healthcare emergency (Cameron et al., 2020; Racine et al., 2021; Salehi et al., 2020). Notably, in a recent report from our group on a cohort of 281 mothers, the rates of clinically relevant symptoms of depression and anxiety during the pandemic were 26 and 32%, respectively (Grumi et al., 2021).

Another well-documented consequence of maternal reduced mental health in the post-partum period is an increase in parenting stress (Larkin & Otis, 2019). This pattern has been defined as the maternal difficulty in adapting to the parental role, possibly due to an overload of perceived requests arising from the demands of the parental role and the sense of not having the resources to absolve them, resulting in psychological distress and depressive states (Gray et al., 2012; Phetrasuwan & Shandor Miles, 2009). Parenting stress is multi-dimensional and only partly depends on maternal psychological adjustment to the transition to motherhood; rather, it includes two relevant dimensions related to caregiving burden and the presence of infants’ difficult, temperamental traits. The different subdimensions of parenting stress largely contribute to the quality of the post-natal caregiving environment, and they may contribute to the association between maternal mental health and infants' developmental outcomes. For instance, Sheinkopf et al. (2006) have reported that the link between neonatal characteristics and temperament might be moderated by maternal parenting stress in a large cohort of at-risk infants exposed to prenatal stress. Among the dimensions of parenting stress, the presence of a dysfunctional or problematic interactive pattern between mother and infant, such as lack of responsiveness, passivity or intrusiveness, low expression of positive affect, withdrawal or avoidance, is of critical concern (Reck et al., 2004; Sheinkopf et al., 2006). As for the COVID-19 emergency, a Chinese study has shown that levels of maternal parenting stress dramatically increased from before to during the pandemic (Tchimtchoua Tamo, 2020). Additionally, post-natal maternal parenting stress has been a significant variable involved in the association between maternal pandemic-related anxiety and later infant temperament at 3 months in Italian mothers (Provenzi et al., 2021).

Home-visiting programs may be beneficial, as they provide a supportive intervention that can increase mothers’ sense of self-confidence and competence as a parent, thus protecting them from developing severe affective symptoms and parenting stress during the first months after delivery (Liu et al., 2012). Despite the existence of different methodological approaches to home-visiting, previous research documented positive effects on maternal health and child development for all of them. For example, a recent study found that a home-visiting program involving first-time mothers at risk, from three to 12 months after their child’s birth, reduced depression, anxiety, and parenting stress (Vismara et al., 2020). Moreover, a review of 21 relevant studies outlined significant improvements to the development and health of young children as a result of home-visiting (Peacock et al., 2013). In particular, the visits prevented child abuse in some cases, and in other instances, developmental benefits concerning cognition, behavioral problems, and, less consistently, language skills. Notably, previous research has mainly focused on the effects of home visits in mothers with moderate or severe depressive symptoms. The study of home-visiting effects in mothers with clinical risk for anxiety disorder is less documented. At the same time, such programs may be especially needed during a healthcare emergency such as the COVID-19, characterized by reduced social support and increased health-related worries in pregnant women (Williams et al., 2021).

In the present study, we report on an Italian cohort of mothers who were in the second or third trimester of pregnancy during the initial outbreak of the COVID-19 pandemic in Italy. The enrolled mothers retrospectively rated the severity of their anxiety symptoms at delivery, reported if they had received at least one home-visiting session from birth to 3 months of their infant’s age, and provided a quantitative estimate of parenting stress at their infant 3-month-age. The main goal was to assess significant differences in the parenting stress levels reported by mothers who received and those who did not receive home-visiting interventions during the pandemic. We hypothesized that mothers who received home visits would disclose less parenting stress than counterparts who did not participate in home-visiting programs. Moreover, we further explored this association controlling for the presence of clinically relevant anxiety levels at delivery. In the absence of previous data, no direction was hypothesized for the findings, and this second aim was exploratory.

## Methods

### Participants

This study is part of the longitudinal and multi-centric *Measuring the Outcomes of Maternal COVID-19-related Prenatal Exposure (MOM-COPE)* research project (Provenzi et al., 2020). Eligible subjects were women in their second or third trimester of pregnancy during the months of the COVID-19 outbreak in northern Italy. No screening on postpartum mood disorder risk was performed. Rather, the opportunity of home-visits was handed to all women at the end of the antenatal classes. They were enrolled between May and October 2020 from ten neonatal units of territorial hospitals located in large provinces of northern Italy (e.g., *Pavia, Piacenza, Lodi, Brescia, Cremona, Milano*). At the time of the present analyses, 263 women were contacted, and 216 (82.1%) women accepted to participate in the study. Mothers were included if at least 18-year-old, in the absence of prenatal and postnatal diseases or injuries, if they delivered at term (i.e., from 37 + 0 to 41 + 6 gestational age), and if they tested negative for COVID-19 at delivery. Mothers were first contacted at antepartum classes or immediately following the post-partum period. The attrition rate between delivery and 3-month data collection time points was 18.1%, as 39 mothers did not provide complete data for the present analyses. Consistently, a sample of 177 women was available for the present study. The study was approved by the Ethics Committee of *Pavia* (protocol ID: 20200037366; ClinicalTrials.gov Identifier: NCT04540029) and the participating hospitals. All mothers provided informed consent to participate in the study.

### Procedure

Socio-demographic (i.e., maternal age, educational level, and occupational status) and neonatal data (i.e., sex, mode of delivery, gestational age, birth weight, head circumference, length, Apgar score at 5 min) were obtained from medical records. Within 48 h from delivery, the mothers filled in a self-report questionnaire rating their anxiety symptoms, namely the state subscale of the State-Trait Anxiety Inventory (STAI-Y) (Pedrabissi & Santinello, 1989; Spielberger, 1989). The theoretical range for the STAI-Y continuous raw score is between 20 and 80, and a score of 40 is a reliable cut-off for clinically relevant anxiety risk (Julian, 2011). The Cronbach’s Alpha was .94. At the 3-month age of their infants, mothers further reported on their concurrent parenting stress by filling in the Parenting Stress Index Short Form, PSI-SF (Abidin, 1983; Guarino et al., 2008). This questionnaire includes 36 items that provide a global score of parental stress and three subscales' scores: (1) Parental Distress (PD): evaluates distress experienced in the parental role, directly related to the demand of the role or resulting from personal factors (e.g., conflicts with the partner, depression, lack of social support); (2) Difficult Child (DC): assesses whether parents perceive controlling their children in terms of their behavioral traits as easy or difficult (measuring the child’s self-regulatory abilities as perceived by the parent); (3) Parent–Child Dysfunctional Interactions (PCDI): indexes parents’ perception of whether their child meets their expectations or not, and how satisfied they are in the relationship with their own child; (4) Total Stress scale: obtained from the sum of the three subscales. It indicates the overall degree of stress that parents experience in their role as parents. The Cronbach’s Alpha was .95. Moreover, mothers retrospectively reported on whether they received home-visiting sessions after delivery further detailing the number of sessions and their satisfaction for the program, including ratings for (a) satisfaction for themes addressed during the sessions, (b) satisfaction for the relationship with the home-visitor, (c) appropriateness of sessions with respect to their expectations.

### Analytical Plan

Mothers who received at least one home-visiting session were compared to counterparts who did not receive home-visiting for the total and subscale scores of the PSI-SF by means of independent-sample *t*-tests. The chi-squared test was used to compare the distribution of clinically relevant anxiety symptoms between the two groups. The association between anxiety levels at delivery (identified by the STAI-Y questionnaire) and parenting stress in the whole group was assessed by means of bivariate correlation; the same analysis was repeated separately for mothers who received and those who did not receive home-visiting sessions. All analyses were conducted using IBM SPSS 27 for Windows, setting *p* < 0.05.

## Results

Participants’ descriptive statistics are reported in Table 1.

**Table 1:** Description of the study sample

	Home-visiting							
	No (n = 123)				Yes (n = 54)			
	Min	Max	Mean	SD	Min	Max	Mean	SD
Apgar at minute 5	8.00	10.00	9.91	0.35	8.00	10.00	9.78	0.46
Gestational age, weeks	37.00	42.00	39.91	1.03	37.00	42.00	39.46	1.04
Birth weight, grams	2480.00	4345.00	3331.55	370.56	2430.00	4260.00	3336.41	453.98
Neonatal length, cm	32.00	56.00	46.50	7.29	33.00	55.00	49.04	4.92
Neonatal head circumference, cm	30.00	53.00	38.03	6.98	32.00	49.00	35.43	3.91
Maternal age, years	22.32	52.63	34.85	4.83	22.65	44.17	33.84	4.37
Maternal education, years	5.00	23.00	14.84	3.51	8.00	22.00	15.30	3.57

	N	%	N	%
Infant sex				
Females	64	52.0	26	48.1
Males	59	48.0	28	51.9
Delivery				
Eutocic	78	63.4	30	55.6
Operative	7	5.7	6	11.1
Caesarean	24	19.5	15	27.8
Exclusive breastfeeding (birth)				
Yes	83	67.5	34	63.0
No	40	32.5	20	37.0
Exclusive breastfeeding (3 months)				
Yes	61	49.6	28	51.9
No	62	50.4	26	48.1
Maternal occupational status				
Employed	107	87.0	50	92.6
Unemployed	15	12.2	4	7.4

Fifty-four (31%) mothers received at least one home-visiting session. Of these mothers, thirty-nine received only one session (72%), nine received two sessions (17%), and six received three or more visits (11%). In the whole sample, forty-eight mothers (27%) reported anxiety levels above the clinical risk cut-off. The percentage of clinically relevant anxiety symptoms did not show a statistically significant difference between mothers who received home-visiting (n = 13, 24%) and those who did not (n = 35, 28%), *χ*^*2*^(1) = .364, *p* = .546. The maternal subjective ratings of appreciation for the single aspects of home-visiting sessions are reported in Table 2.

**Table 2:** Maternal subjective ratings from 1 (low) to 6 (high) of appreciation for the home-visiting sessions

	Mean	SD
*Satisfaction of general expectations*		
Feeling helped in improving breastfeeding	5.04	1.6
Feeling helped in understanding the child's behavior	4.65	1.64
Receive information on development	4.88	1.49
Receive information on how to promote the infant's physical well-being	4.8	1.48
Receive information on how to promote the infant's emotional well-being	4.53	1.62
Feel comfortable as a parent	4.82	1.45
Feel more secure as a parent	4.82	1.51
*Perception of the relationship with the operator*		
I felt heard	5.52	1.14
I felt supported	5.5	1.18
I had the opportunity to ask questions	5.69	0.93
I felt entitled to express doubts	5.5	1.09
I felt I could trust	5.57	1.14
I did (not) feel judged	5.35	1.54
It made me feel less alone	4.57	1.84

The overall satisfaction was high for both general expectations (with a mean rating of 4.8 out of 6, *SD* = 0.08) and the relationship with the operator (mean rating of 5.4 out of 6, *SD* = 0.31).

**Fig. 1:**
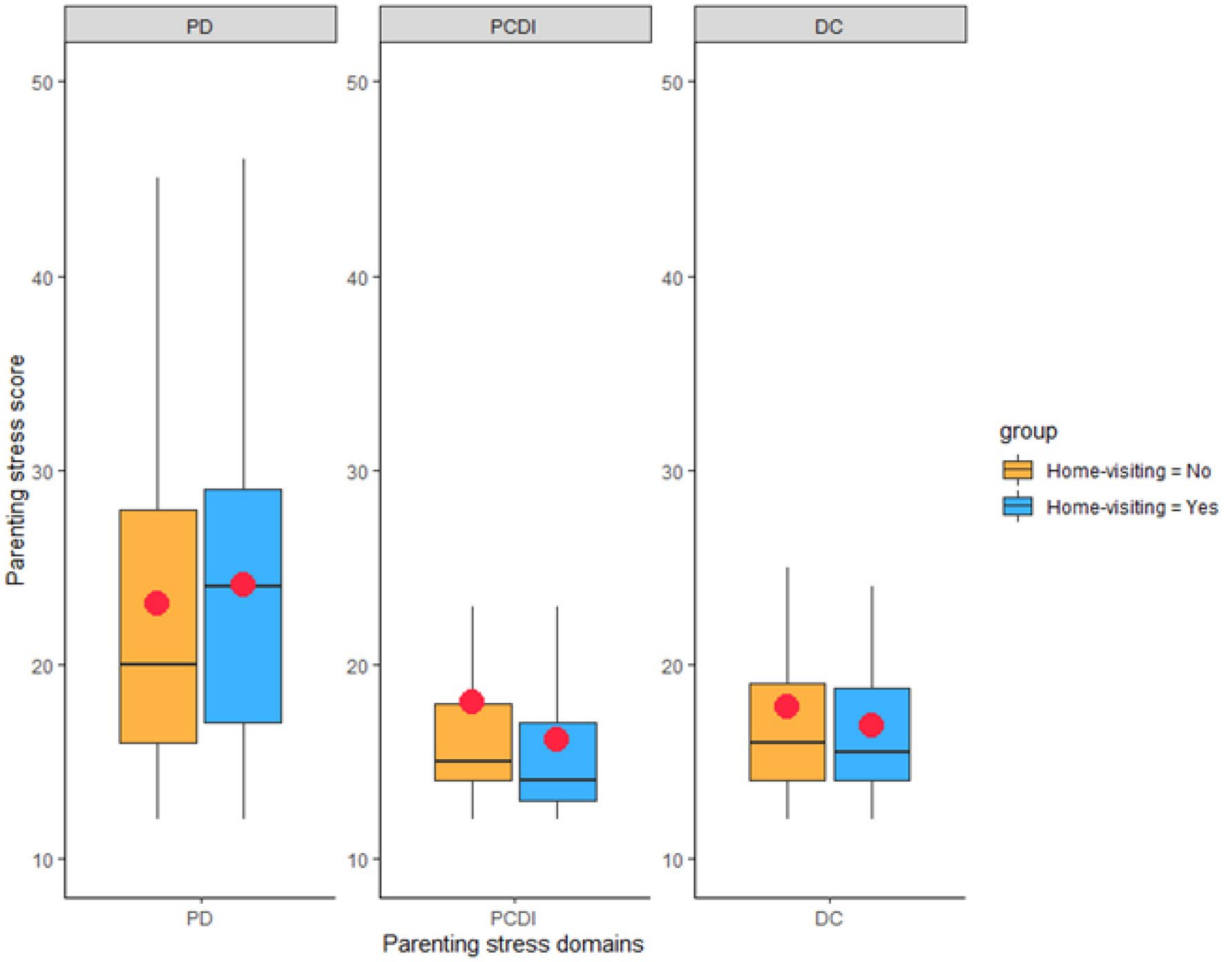
Parenting stress scores in mothers who received at least one home-visiting session and in counterparts who did not receive any home-visiting session. For each boxplot, red dots represent mean values, the horizontal line represents the median value, whiskers indicate min and max values, and box segments the distribution between first and third quartiles (Color figure online)

Anxiety levels at birth measured through the STAI-Y did not differ between the two groups of mothers (all *ps* > 0.05). 3 months after delivery, mothers who received at least one home-visiting session reported lower stress in the PCDI subscale of the PSI-SF compared to counterparts who did not receive home-visiting, *t* (175) = 2.11, *p* = .037 (Fig. 1).

**Fig. 2:**
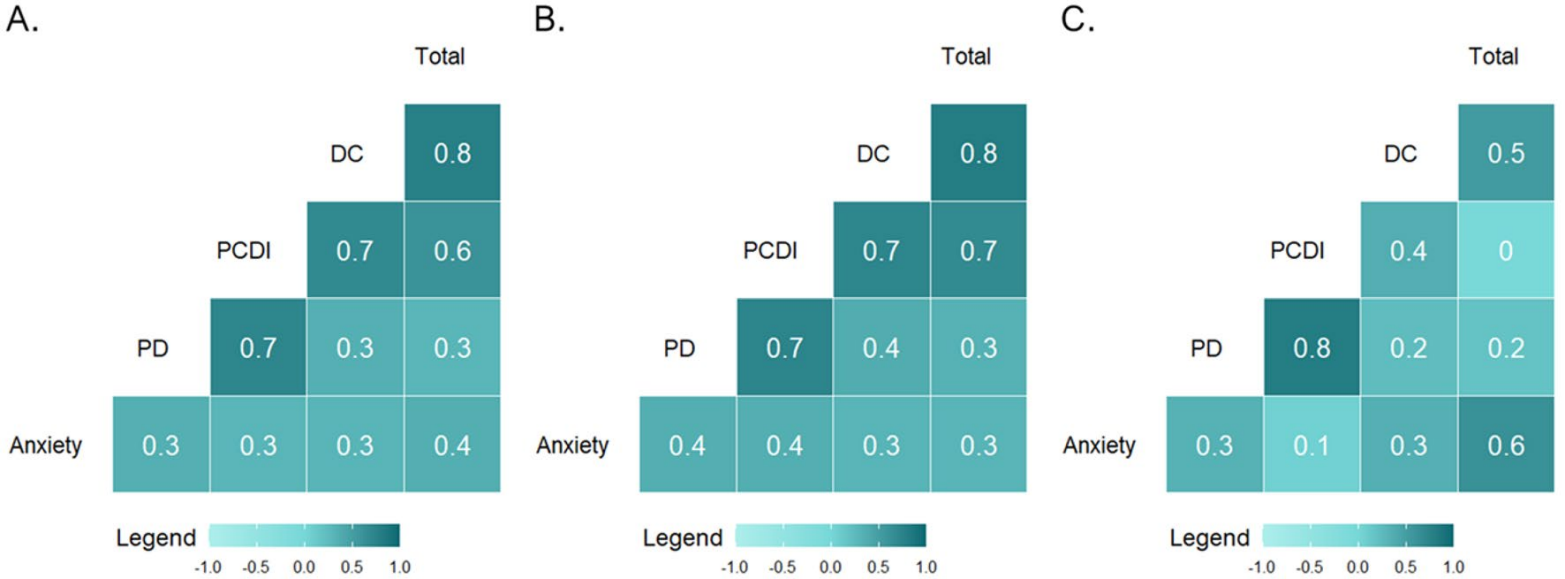
Correlation matrix for maternal anxious symptoms after delivery and parenting stress scores at 3 months in the whole sample (**A**), in mothers who received no home-visiting (**B**), and in mothers who received at least one home-visiting session (**C**)

No statistically significant differences emerged for the other PSI-SF subdomains and Total Stress score between mothers who received and those who did not receive home-visiting sessions. The correlation between the continuous scores of maternal anxiety and each of the parenting stress subdomains was then analyzed, and the scores are reported in the matrix in Fig. 2.

**Fig. 3:**
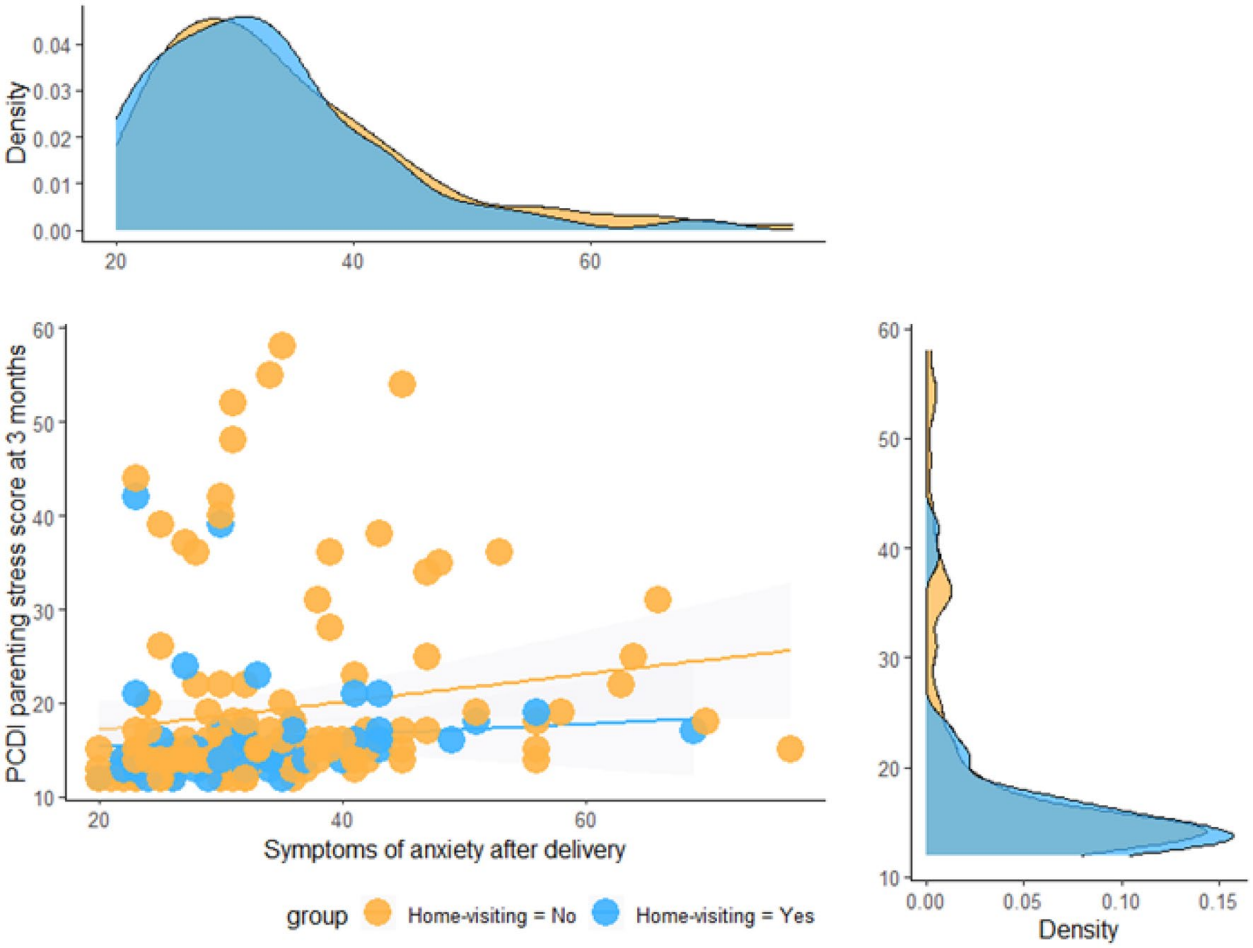
Linear association between maternal symptoms of anxiety after delivery and parenting stress at 3 months in mothers who received at least one home-visiting session and in counterparts who did not receive any home-visiting session

In the whole sample, significant associations emerged between maternal anxious symptoms after delivery and 3-month parenting stress for all subscales of the PSI-SF (all *p*s < .05). In general, higher anxiety levels at delivery corresponded to higher parenting stress reported after 3 months. Notably, when the correlation between anxiety levels at delivery and parenting stress was assessed separately for mothers who received and those who did not receive home-visiting sessions, the correlation between anxiety and PCDI was significant, *r* = .36, *p* < .001, in the group of mothers who did not receive home-visiting whereas it was not for mothers who received at least one session, *r* = .058, *p* = .676 (Fig. 3).

## Discussion

This study aimed to identify the possible beneficial effects of home-visiting programs in reducing parenting stress in mothers and reducing the risk of increased parenting stress in mothers who showed high levels of anxiety after delivery during an unprecedented global traumatic experience such as the COVID-19 pandemic.

Our results showed that home-visiting was associated with lower parental stress in the subscale of Parent–Child Dysfunctional Interactions (PCDI), while no decrease in stress scores was observed for the other subdomains. Therefore, the beneficial effects of post-natal home visits were observed for a specific component of parenting stress that reflects the dyadic interactive aspects of the mother-infant relationship. In contrast, the other two subdomains (i.e., Parental Distress and Difficult Child) provide quantitative estimates of maternal stress related to individual sources. This finding can be better understood considering that the primary goal of home-visiting sessions is not to reduce individual maternal distress or to provide behavioral interventions for child behavior regulation. Rather, home visits are directed explicitly at increasing maternal feelings of self-confidence, competence as a parent, and perception of social support (Liu et al., 2012). As such, the fact that this early preventive support might be effective in reducing the risk of stress arising from potential issues in parent-infant interaction is not surprising and extend previous evidence on the benefit of home visiting programs (Castel et al., 2016; Kaaresen et al., 2006; Kiani et al., 2021) to the current pandemic time. Indeed, it should be highlighted that in rating their satisfaction towards the intervention, all mothers reported that they felt helped in breastfeeding, understanding their child's behavior, obtaining information about physical and emotional development, and feeling more competent as a parent. Interestingly, even higher rates were given to those aspects that involved the relationship with the care provider, such as feeling heard, supported, having the opportunity of asking questions and expressing doubts. Mothers felt that they were less alone, not judged and that they had someone they could trust (Table 2). Mirroring the findings that showed how the dyadic interactive aspects of mother-infant relationship were the ones that benefitted more from the intervention, we confirm that the social connections and support are the core of a heightened well-being perception in mothers who were exposed to social separations during pregnancy. Home-visiting may provide a social resource in the face of the isolation and trauma experienced by expecting mothers during the COVID-19 pandemic (Provenzi & Tronick, 2020).

It should be highlighted that parenting stress was found to be significantly correlated with maternal anxiety in this cohort. Nonetheless, the statistical significance was confirmed only for the latter when controlling for this association in the home-visiting and non-home-visiting groups. As the two groups did not differ in the prevalence of clinically significant anxiety, this finding further supports the hypothesis that home visiting programs should be specifically beneficial for mothers who present high levels of anxiety after delivery. In other words, home-visiting appears to be a source of protection for maternal mental health and the development of a nurturing caregiving environment, particularly during a global healthcare emergency. As delivering home-visiting programs to mothers during the first 3 months may act as a buffer between maternal anxiety and parenting stress, these preventive interventions are largely warranted to be offered to mothers regularly (Kersten-Alvarez et al., 2010; Rotheram-Borus et al., 2014).

A limit of the present study is that most mothers received only one home-visiting intervention (in the 72% of cases). The home-visiting programs are generally more beneficial with the increase in the number of visits (Nygren et al., 2018). The lower time dedicated to building parenting skills and confidence might be responsible for the lack of a general decrease in stress perceived by the mothers in our sample. Moreover, we reported an Italian cohort of mothers only. Thus, the generalization of our results might not always be possible, as different cultures and social structures greatly influence perceived support and stress (Campos & Kim, 2017). Keeping the focus on providing information about the child’s physical health and child development, other than supporting the parent–child relationship, is critically important. Finally, the satisfaction about the home-visiting program was reported retrospectively, and parenting stress was measured through a self-report scale. Having direct measures of such aspects might increase sensitivity in detecting changes in stress and allow a more controlled analysis of what home visits contribute to these changes.

## Conclusions

In conclusion, the challenges of facing a pandemic imply a significant amount of additional stress, especially in such crucial phases as pregnancy, delivery, and the first post-natal months. Importantly, for those cases where, due to an individual predisposition or especially detrimental conditions, anxiety is predominant, a heightened risk of parental stress is indeed present. This is precisely where a home-visiting program can be fundamental towards effectively reducing the risks for both mothers and infants. Future clinical implications might consider using home-visit programs as an essential tool to improve parent–child interactions during a pandemic. Indeed, the COVID-19 emergency requires us to bridge the existing gap between research and clinical practice implementing, amongst several aspects, smart diagnostics, effective prevention, and compassionate communication (Horesh & Brown, 2020). We believe that screening anxiety levels in mothers after delivery might help detect those cases of heightened risk where implementing house-visiting programs that limit COVID-19- specific trauma in the first year of infants’ life would be of foremost importance.

## Data Availability

The data included in this study have been made available on a permanent third-party archive (Zenodo) at this link: 10.5281/zenodo.7104124; further requests can be sent via email to the corresponding author.

## Code Availability

No custom code was used.
